# Prevalence of Gastrointestinal Parasites in the Frugivorous and the Insectivorous Bats in Southcentral Nepal

**DOI:** 10.1155/2020/8880033

**Published:** 2020-12-12

**Authors:** Roshan Babu Adhikari, Mahendra Maharjan, Tirth Raj Ghimire

**Affiliations:** ^1^Third Pole Conservancy, Wildlife and Eco-health, Bhaktapur, Nepal; ^2^Central Department of Zoology, Tribhuvan University, Kathmandu, Nepal; ^3^Animal Research Laboratory, Faculty of Science, Nepal Academy of Science and Technology (NAST), Lalitpur, Nepal

## Abstract

Bats are the only active flying placental mammals and are traditionally classified into mega- and microbats, which are, respectively, herbivorous and insectivorous in feeding habit. Though deforestation, habitat destruction, natural calamities, illegal hunting, and climate changes are the challenging threats for bats, the role of existing gastrointestinal (GI) parasites have not been evaluated yet in Nepal. Thus, the current study aims to determine the prevalence of GI parasites in bats from the Shaktikhor area at the Chitwan district of Southcentral Nepal. From July 2018 to February 2019, a total of 60 fecal samples of bats (30 from frugivorous bats and 30 from the insectivorous bats) were collected. These samples were preserved at 2.5% potassium dichromate solution. The fecal examination was carried out by the direct wet mount, concentrations, acid-fast staining, and sporulation techniques. Overall results showed the prevalence rate of 80% GI parasites. The parasites detected in the insectivorous bats were Ascarid spp., Capillarid sp., *Cryptosporidium* sp., *Eimeria* spp., *Entamoeba* sp., *Giardia* sp., *Hymenolepis* spp., *Isospora* sp., Oxyurid sp., Strongyle, and *Strongyloides* sp. In contrast, *Eimeria* sp., *Entamoeba* sp., and *Hymenolepis* sp. were detected in the frugivorous bats. Based on a wide diversity of parasite richness and parasitic concurrency measured by the prevalence rates, we suggest that GI parasitism might be a threatening factor in the insectivorous bats in the current study area.

## 1. Introduction

Bats belonging to the order Chiroptera are the only active flying true placental mammals of the animal kingdom. Chiroptera is the second largest order of mammals (after the rodents) with cosmopolitan distribution [1, 2]. Bats are traditionally classified into the megabats and microbats [3–5]. Megabats include flying foxes and the old-world fruit bats, which are usually herbivores and consume fruits, flowers, leaves, nectar, and pollens [3, 5, 6]. In contrast, microbats are mostly insectivorous in feeding habits; although, few of these species may feed on blood, fruits, nectars, pollens, and vertebrates [3, 7–10].

It has been estimated that more than 1300 species of bats are reported in the world [1, 8]. However, many species are threatened with extinction globally, and more than 280 species are categorized as endangered, vulnerable, or near threatened by the IUCN Red List [11]. In the context of Nepal, there are a total of 54 bat species belonging to seven different families indicating this Himalayan nation to be one of the rich countries in their diversities [12]. However, deforestation, habitat destruction due to the operation of road construction projects and natural calamities, illegal hunting, and climate changes exist as challenging threats for these mammals [13]. Among these threatening factors, diseases might be critical because these mammals play roles as pathogen carriers, reservoirs, and transmitters in nature. The disease-causing pathogens are viruses, bacteria, fungi, and parasites, which can be life threatening in humans and animals. It should be noted that several species of gastrointestinal (GI) protozoa, trematodes, nematodes, and cestodes have been predominantly reported from the bats of various geographies, and they may remain as one of the major threats for their lives [14–21]. Moreover, infected bats act as definitive or intermediate or a paratenic host for many protozoan, trematode, cestode, and nematode parasites [21]. In these situations, feeding behavior, biological, and ecologic diversity of bats might play a critical role in the host-parasite interactions and parasitism [21]. However, the study of these parasitic faunae in bats has been still at virgin state in Nepal. Besides, the association of GI parasitism based on the feeding ecology of bats has not been determined and compared so far. Thus, in this study, we have investigated the prevalence of GI parasitic species in the frugivorous and the insectivorous bats found in Chitwan, the Southcentral part of Nepal.

## 2. Materials and Methods

### 2.1. Study Area

**Figure 1:**
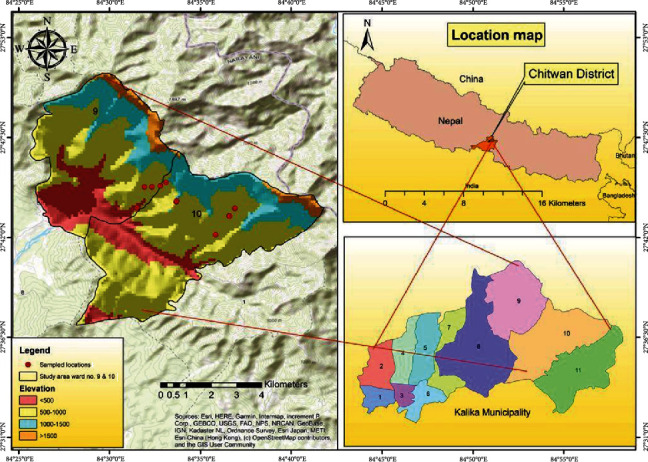
Map of the study area showing the locations of sample collections.

The current study was conducted in ward no. 9 and 10 of Kalika Municipality, the commonly called Shaktikhor area (251 m to 1003 m above sea level, asl) (Figure 1). The geographic locations range from (27.69544–27.73472) N to (84.57159–84.65498) E in Chitwan district, in the Southcentral part of Nepal. It is 182 kilometers (kms) away from the capital city and is linked to the East-West highway by road up to the foothills. The climate is tropical to subtropical, with an average annual temperature of 29.30°C during summer and 9.4°C during winter. Similarly, the yearly average rainfall is 1993 mm [22]. The vegetation of this area includes lowland Sal forest, hill Sal forests, tropical riverine forest, tropical mixed broad-leaved forest, and subtropical mixed forest [23], and a total of 13 species of birds, eight species of mammals, and six species of reptiles have been reported to inhabit this area according to the Environmental Impact Assessment done in 2019 [22].

### 2.2. Sample Collection, Preservation, and Transportation

**Figure 2:**
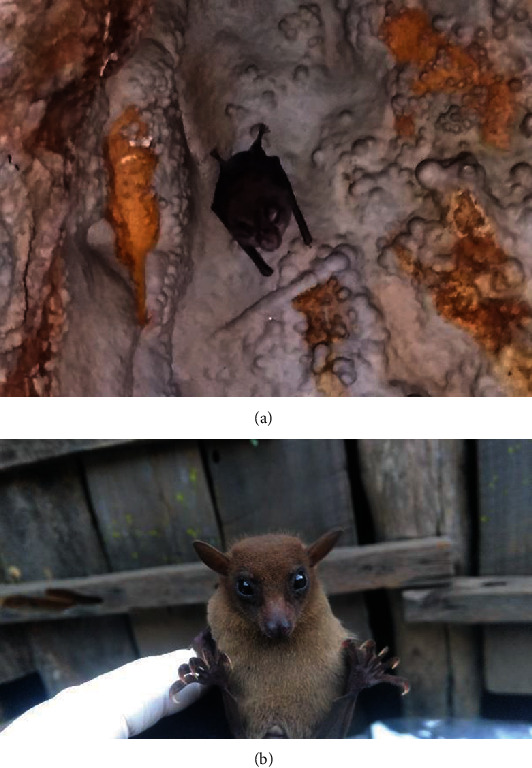
(a) Photograph of an insectivorous bat in the tunnel. (b) Photograph of a frugivorous bat.

A total of 60 fecal samples (30 from the frugivorous and 30 from the insectivorous bats) were collected from July 2018 to February 2019 from the study area. The frugivores included *Rousettus leschenaulti* and *Eonycteris spelaea* and insectivores included *Rhinolophus macrotis*, *Rhinolophus pusillus*, and *Rhinolophus pearsonii* [13] (Figure 2). For fecal sample collection, 30 frugivorous bats were captured using the net at night time from five different spots in the butter tree (*Diploknema butyracea*) forest, and anal swabbing was performed with the help of cotton buds. In the context of the insectivorous bats, a total of 30 clean white plastic were overlaid on the floor of five different caves (six plastics per cave) just below their roosts in the morning. The fecal samples that fell down the plastic were collected with the help of forceps in the evening. Quality control during sample collection was performed by observing the absence of other mammals inside the wet and dark caves. The collected samples were immediately preserved at 2.5% potassium dichromate solution in 20 mL sterile vials. They were transported to Animal Research Laboratory (ARL) of the Nepal Academy of Science and Technology (NAST) and further stored at 4 degrees (°) Celsius temperature.

### 2.3. Laboratory Processing and Examination

The fecal samples were macroscopically examined for the presence of blood, mucus, segments of cestodes, as well as whole adult nematodes and microscopically examined by the techniques based on the literatures, explained previously [24, 25].

#### 2.3.1. Direct Wet Mount Technique

One to two drops of carefully stirred fecal samples were put in the slide with the help of a plastic dropper. The samples were observed directly at 2.5% (*w*/*v*) potassium dichromate, Gram's iodine stain, and Giemsa's stain (1/15).

#### 2.3.2. Saturated Salt Floatation Technique

About two grams (gms) of the fecal samples were thoroughly mixed in a 13 milliliter (mL) normal saline (0.9% *w*/*v*) solution and filtered with the help of a tea strainer. The solution was poured into a 15 mL conical centrifuge tube and proceeded to centrifuge (1200 revolutions per minute, rpm for 5 minutes). After discarding the supernatant, 12 mL of salt solution (45% *w*/*v*) was added and proceeded to centrifuge (1200 rpm for 5 minutes). Then, few drops of salt solution (45% *w*/*v*) were added in the tube to fill it, and a coverslip was placed on the mouth of the tube. After 10 minutes, the coverslip was carefully removed and put on the glass slide with or without Lugol's iodine for microscopic observation at 100x and 400x total magnifications.

#### 2.3.3. Sedimentation Technique

About two gms of the fecal samples were thoroughly mixed in 13 mL normal saline (0.9% *w*/*v*), were filtered with the help of a tea strainer into a 15 mL centrifuge tube, and were proceeded to centrifuge (1200 rpm for 5 minutes). Then, the supernatant was discarded, and one to two drops of the sediment was put on a glass slide. Gram's iodine and Giemsa's stain (1/15 dilutions) were differently used in the deposits for the microscopic examinations at 100x and 400x total magnifications.

#### 2.3.4. Acid-Fast Staining

About one gm of the *Cryptosporidium*-positive sediments, 10% 10 mL formalin, and 4 mL ethyl acetate were taken in a 15 mL centrifuge tube and proceeded to centrifuge (1200 rpm for 5 minutes). Then, the supernatant was discarded, and the sediments were used to prepare thin smears. This smear was allowed to dry at room temperature and then fixed in the absolute methanol for 2 minutes. The smear was stained with carbol fuchsin for 15 minutes at room temperature and then washed with distilled water followed by destaining with acid alcohol, and finally rinsed with distilled water. The smear was further restained with malachite green for one minute, followed by washing with distilled water, and allowed to dry at room temperature. The dry slide was observed at 1000x total magnification using immersion oil.

#### 2.3.5. Sporulation Assay

About two gms of coccidian positive samples were incubated at equal volumes of 2.5% potassium dichromate at 28°C ± 1 temperature in an incubator for sporulation assays. Then, using the floatation method, the sporulation states were observed at each 24 hours interval under the microscope [26–28]. The presence of oocysts of *Eimeria* spp. and *Isospora* sp. was confirmed by their respective spore formulas as 0.4.2 and 0.2.4, as reviewed previously [29].

#### 2.3.6. Parasite Identification

All the fecal parasites were carefully observed under a light microscope (Optika Microscopes Italy, B-383PLi) at a total magnification of 100x, 400x, and 1000x. Photographs were taken by the camera (SXView 2.2.0.172 Beta (Nov 6, 2014) Copyright (C) 2013-2014) accompanied by the microscope. The size of the parasites was assessed by using ImageJ 1.51k (National Institute of Health, USA), and identification was carried out based on various literature [30–36].

### 2.4. Data Analysis

Data were expressed as numbers of positive samples as well as prevalence rates in the table using Microsoft Word. Prevalence rates were calculated by dividing the number of GI positive samples (total or particular species) by the total number of samples observed [24]. We used the GraphPad Software (Prism 5 for Windows Version 5.00 @ 1992–2007 GraphPad Software, Inc). We applied Fisher's exact test (two-sided) to assess *p* values by comparing the prevalence of specific GI parasitic groups between the frugivorous bats and the insectivorous bats. Statistical significance was considered at the 95% confidence interval (*α* = 0.05, *p* < 0.05).

## 3. Results

**Figure 3:**
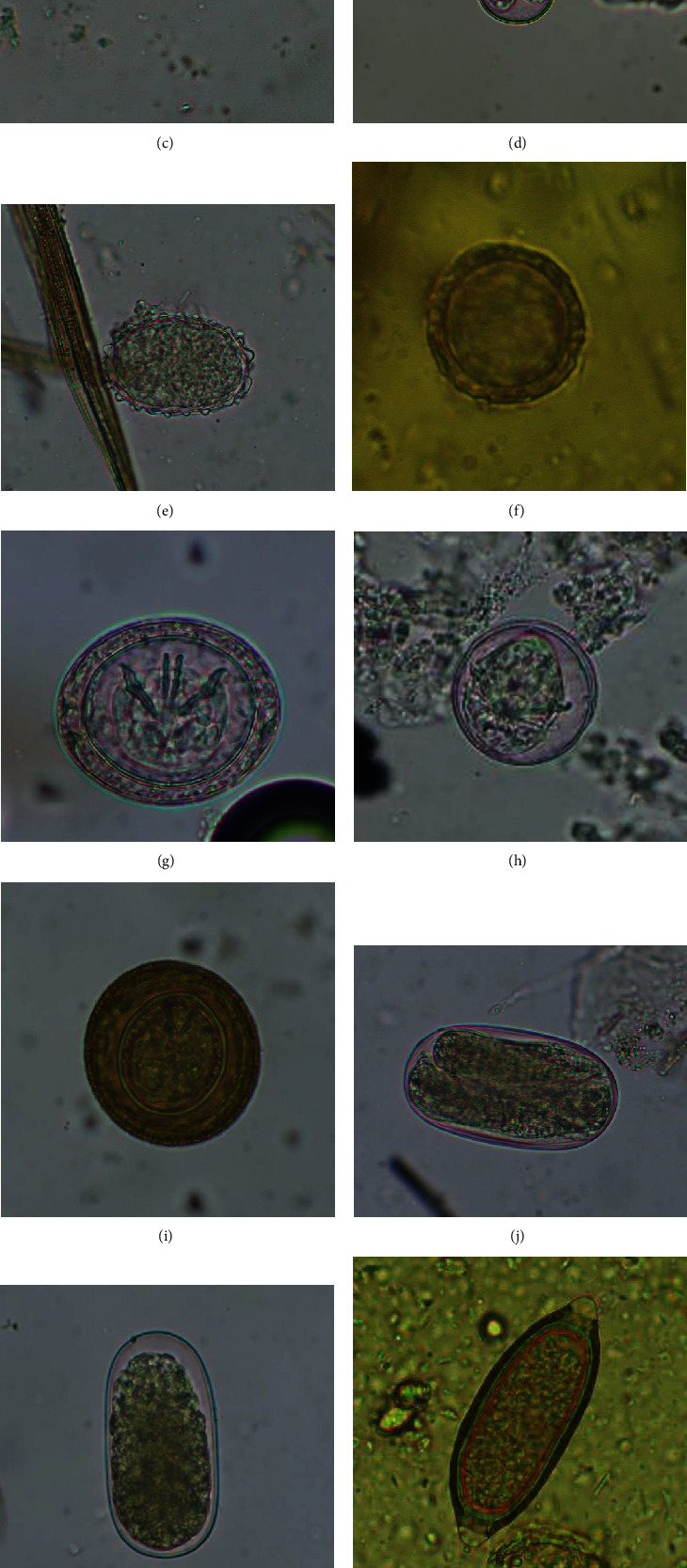
Photomicrographs of various parasitic species. (a) Oocyst of *Eimeria* sp. (i) (20 × 14 *μ*m), 400x, direct wet mount at Gram's iodine stain, in insectivorous bat. (b) Oocyst of *Eimeria* sp. (ii) (17 × 15 *μ*m), 400x, after flotation technique at Giemsa's stain, in insectivorous bat. (c) Cyst of *Entamoeba* sp. (11 × 11 *μ*m), 400x, direct wet mount at Lugol's Iodine stain, in insectivorous bat. (d) Oocyst of *Isospora* sp. (25 × 23 *μ*m), 400x, after flotation technique, in insectivorous bat. (e) Egg of Ascarid sp. (54 × 36 *μ*m), 400x, after sedimentation technique at Giemsa's stain, in insectivorous bat. (f) Egg of *Toxocara* sp. (50 × 49 *μ*m), 400x, direct wet mount at 2.5% potassium dichromate, in insectivorous bat. (g) Light purple-colored egg of *Hymenolepis* sp. (52 × 43 *μ*m), 400x, after flotation technique, in insectivorous bat. (h) Egg of *Hymenolepis* sp. (44 × 43 *μ*m), 400x, after flotation technique, in frugivorous bat. (i) Brown-colored egg of *Hymenolepis* sp. (65 × 62 *μ*m), 400x, after flotation technique in insectivorous bat. (j) Egg of *Strongyloides* sp. (87 × 46 *μ*m), 400x, after sedimentation technique at Gram's iodine stain, in insectivorous bat. (k) Egg of Strongyle (83 × 41 *μ*m), 400x, after flotation technique, in insectivorous bat. (l) Egg of Capillarid sp. (67 × 25 *μ*m), 400x, direct wet mount at 2.5% potassium dichromate, in insectivorous bat. (m) Egg of Oxyurid sp. (93 × 36 *μ*m), 400x, after flotation technique, in insectivorous bat.

In the current study, out of 60 fecal samples, 80% (60% in the frugivorous and 100% in the insectivorous bats) were positive for at least one GI parasitic species. The sensitivity of different tests gave different results, for example, direct wet mount, sedimentation, and flotation techniques detected GI parasites in 61.7% (37/60) with seven species, 73.3% (44/60) with nine species, and 76.7% (46/60) with nine species, respectively. The overall prevalence of protozoan and helminth parasites was 70% and 50%, respectively. In this context, the prevalence of protozoa was double (93% versus 46.7%) (*p* < 0.0001), and that of helminths was four times greater (80% versus 20%) (*p* < 0.0001) in the insectivorous bats compared to the frugivorous bats. The prevalence of specific GI parasites in frugivores was *Entamoeba* sp. (40%), *Eimeria* sp. (13.3%), and *Hymenolepis* sp. (20%). In contrast, the insectivores possessed *Eimeria* spp. (83.3%), Strongyle (56.7%), *Hymenolepis* spp. (50%), *Entamoeba* sp. (30%), *Isospora* sp. (16.7%), *Strongyloides* sp. (16.7%), Ascarid spp. (16.7%), *Cryptosporidium* sp. (10%), Oxyurid sp. (6.7%), *Giardia* sp. (3.3%), and Capillarid sp. (3.3%) (Figure 3) (Table 1).

Further, we classified *Eimeria* spp. into six different morphologic forms in the insectivorous bats; however, a single morphotype of this coccidian was present in frugivores. Similarly, in the context of helminths, six species of parasites were found in the insectivorous bats, but only one *Hymenolepis* sp. was detected in the frugivorous bats. In frugivores, the eggs of *Hymenolepis* sp. were light purple (average size ranges: 42–48  *μ*m × 40–46 *μ*m). In contrast, the eggs were light purple as well as dark brown (average size range of 48–66  *μ*m × 39–62 *μ*m) in the insectivores. In the insectivorous bats, a total of three samples were positive for eggs similar to human *Ascaris*, and one sample was positive for eggs similar to *Toxocara pteropodis*. Therefore, we named Ascarid spp. to the *Ascaris*-like and *Toxocara pteropodis*-like eggs detected. Similarly, three morphotypes (size ranges: 58–92  *μ*m × 25–58 *μ*m) of the eggs of Strongyle were identified in the insectivorous bats (Figure 3).

The concurrency of the GI parasitism in the fecal samples was also analyzed. Single infection was higher in the frugivorous bats than in insectivores (46.7% versus 13.3%). In contrast, multiple infections were significantly higher in the insectivorous bats than in frugivores (86.7% versus 13.3%) (*p* < 0.0001). The insectivorous bats contained mixed infections up to five various species, whereas, in frugivores, we found mixed infections only up to two different species (Figure 3) (Table 1). *Entamoeba* showed maximum single (57%) and double (100%) infections in frugivorous bats. In contrast, *Eimeria* showed maximum single (75%), double (86%), triple (100%), quadruplet (66.7%), and pentuplet (100%) infections in insectivorous bats (Supplementary file 1).

## 4. Discussions

To the best of our knowledge, the current study was the first attempt to investigate the prevalence study of GI parasites of bats according to their feeding habit in this Himalayan nation. The current prevalence rate of GI parasites (80%) in bats was lower than the findings from France (100%) [37], Brazil (96.29%) [34], Serbia (88.2%) [20], and South Africa (85.5%) [38]; slightly higher than those recorded from Argentina (78.6%) [39], Nigeria (76.78%) [40], England (76%) [41], and Mexico (72–76%) [42, 43]; and higher than those from the United States (63.6–75%) [15, 44] and Egypt (43.5%) [45]. These differences might be attributed to the application of different sampling techniques in the field, different methods in different laboratories, and climatic scenarios in the various study sites. The current study used the direct wet mount, concentration techniques, acid-fast staining, and sporulation assays that might have produced high positive cases. Besides, few factors like pathogen-harboring nature, colonizing or aggregating behaviors, and species of the bats [46–51] might also differently govern parasitic infectiousness. Except for the report of Lima and colleagues [34] and some experiments involving coccidian morphology [15, 44], most of the studies are based on the histopathologic findings [20, 38, 40–43, 45], and in these contexts, it is not easy to compare our results with their investigations.

The diversity in parasite richness and parasitic concurrency, as measured by the parasitic prevalence, was higher in insectivores than in frugivores. This discrepancy might be explained based on different feeding habits and the landscapes of the habitat used. Landscapes include available diets, roosting sites, water sources, foraging habitats, and shared ecosystems with other animals. Firstly, insectivores usually prefer insects like bees, beetles, caddis flies, cockroaches, crickets, flies, flying ants, grasshopper, mayflies, mosquitoes, moths, termites, and wasps [8, 40, 52]. One or more of these insects are also known to act as intermediate hosts or transport vectors for helminth and or protozoan parasites [14, 53–58]. Secondly, insectivores mostly spend their lives in the caves with high moisture contents, which are essential for the survival and development of the eggs, cysts, oocysts, and larva of the GI parasites [59]. Thirdly, these bats usually spend the full day on roosts that can result in the evaporation and extreme loss of water from their body [60]. Therefore, after coming out of the roosts, they directly visit the water sources and drink water regularly to rehydrate themselves [61]. For foraging and drinking, most insectivores are known to utilize aquatic habitats like canals, farms, urban dams, lakes, streams, rivers, and swimming pools [62–65]. In the study areas, open defecation, nearby water sources, and fields were observed. Also, domestic animals like chicken, goats, cattle, dogs, and pigs of the study areas share the same water sources. They can contaminate them with infective cysts, eggs, oocysts, and larva of GI parasites. In these scenarios, we cannot ignore the possibility of cross-transmission of many parasites; although, further epidemiologic proofs are needed to confirm this opinion. In contrast to these bats, frugivores compensate for the requirement of water from plant/fruit juices and occasionally use the water sources [66, 67]. That is why they are less exposed to parasites.

In this research, compared with the frugivorous bats, the insectivorous bats possessed higher concomitant infections. Similar to our study, mixed infections by protozoa (*Eimeria*, *Entamoeba*, *Giardia*, and *Cryptosporidium*) and by protozoa and helminths (Ancylostomatidae, *Vampirolepis nana*) in Brazilian bats have been predominantly reported [34]. Concomitant infections are the rules rather than the exception [68], impact on the fitness of host as well as the epidemiology of the pathogens in all biological communities [68], and help investigate the role in the emergence of zoonoses [69]. Polyparasitism is the complex interactions among various species, and the outcome of those interactions can be synergistic (positive), antagonistic (negative), or neutral [68]. In positive case, the presence of one pathogen may enhance the infection by other pathogens. In negative case, one pathogen inhibits the infection or reproduction of other pathogens, for example, cats infected by many species possessed lower *Toxocara* loads [70]. In neutral case, there is no influence on infection by other pathogens. Our results of maximum coinfection by *Eimeria* in fecal samples suggest that further studies should be conducted to link this coccidian in GI pathogenesis. Notably, the link of GI parasites in gastroenteritis has not been fully enlightened in a polyparasitized bat host. Thus, rather than single species, the effects of polyparasitism by enteric pathogen communities should be assessed especially in pathologic consequences [70, 71].

It was interesting that *Eimeria* spp. were the predominant species in the insectivorous and overall bats. Their prevalence rate (83.3%) in insectivores was lower than the findings from France (100%) [37] and higher than reported from Europe (80%) [72], the United States (75%) [44], Brazil (74.07%) [34], the United States (63.6%) [15], Japan and North America (3.4%–7%) [31, 73], and Northwestern Arkansas (13%) [15]. Similarly, the prevalence of *Isospora* sp. was 16.7% in the insectivorous bat, and this rate was slightly higher than the finding in the big brown insectivorous bats (*Eptesicus fuscus*) from the United States (4.92%) [35]. Another important coccidian parasite detected in insectivores was *Cryptosporidium* with the prevalence of 10% which was slightly higher than the finding from China (7.7%) [74] and the Philippines (8.8%) [43] confirmed by molecular methods and slightly lower than the results from Brazil (11.11%–16.3%) [34, 75]. This coccidian parasite was also reported from the fecal samples of two insectivorous bats *Pipistrellus pipistrellus* and *Myotis ciliolabrum* via the molecular methods from the USA and Czech Republic [76] indicating these coccidia are predominant in bats.

Regarding Sarcodina, the prevalence of *Entamoeba* sp. was 30% in insectivores and 40% in frugivores suggesting both bats are critical reservoirs for this ameba. This prevalence was lower than that reported from *Molossus molossus*, an insectivorous bat in Brazil (32%), and higher than that reported from two other insectivorous species like *Myotis lavali* (10%) and *Noctilio albiventris* (21.05%) [34]. Moreover, amebic dysentery caused by *Entamoeba histolytica* was firstly reported by techniques similar to ours and molecular assays in *Rhinolophus rex*, an insectivorous bat, from China indicating its pathologic consequences in bats [77].

Interestingly, only one sample (3.3%) of insectivore was positive for *Giardia* sp. which was lower than reported in the similar hosts from Brazil (11.10%) [34], indicating that this flagellate cannot be ignored during diagnosis of GI parasitism in bats.

Among the helminths, the overall prevalence of *Hymenolepis* spp. was the highest and was reported from both types of bats. The current prevalence of this tapeworm in insectivores (50%) was slightly higher than the finding from Brazil (48.14%) [34]. Similar genera have been predominantly reported from insectivores by other studies around the globe [21, 38–40, 78, 79]. Some of these species include secondary hosts like insects in their life cycle. Thus, the current result indicates that parasite transmission is related to the feeding characteristics of the bat hosts, and it is the reason why the insectivorous bats were found to be positive with this cestode. Furthermore, we have reported the same genus of different morphotypes in the frugivorous bats, with a prevalence of 20%. This rate was higher than that reported from Amazonia Brazil (1.49%) in *Artibeus planiros*, a frugivorous bat [80].

In the current study, except for *Hymenolepis* spp., all other helminths like Ascarid spp., Strongyle, Oxyurid sp., *Strongyloides* sp., and Capillarid sp. were reported only from insectivores. We grouped three different morphotypes of nematode eggs into “Strongyle-type,” because, without larval cultures, it is not easy to differentiate them only via the egg morphometry. Many previous histologic studies of GI tracts of the insectivorous bats from various geographies were conducted. They reported the presence of the adults of different Strongyles like *Histostrongylus coronatus*, *Macuahuitloides inexpectans, Molinostrongylus ornatus*, *Parahistiostrongylus octacanthus*, *Strongylacantha glycyrrhiza*, *Torrestrongylus tetradorsalis*, and *Bidigiticauda serrafreire* [20, 21, 38, 40, 42, 80–82]. This evidence indicates the predominance of a wide variety of these nematodes.

There were two morphotypes of eggs of Ascarid spp. in the current insectivorous bats with the prevalence rates of 16.7%, which was higher than the finding from Brazil [34]. This roundworm species was also reported in a few research findings [83, 84]. Although we did not report these nematodes from frugivores, previous studies reported the presence of *Toxocara pteropodis* in frugivore bats from Australia [85], Palm Island (25%) [86], and Sri Lanka (13%) [87].

It was notable that in insectivores, we reported *Strongyloides* sp. with a prevalence of 16.7%, which was slightly higher than reported from Brazil (9.25%) [34]. Another nematode Capillarid sp. was reported to be present in 3.3% insectivores, and this rate was similar to those reported from Brazil (1.49%–3.7%) [34, 80, 88] and was lower than from Nigeria (18.44%) [40]. This nematode was also reported in 2% frugivorous bats from Amazonian Brazil [67]. Interestingly, we found eggs of oxyurid nematodes in 6.7% of the insectivorous bats and are the first record in published peer-reviewed journals. The presence of this nematode may suggest two possible hypotheses; firstly, oxyurids are natural in bats. Secondly, bats may acquire them via cross-transmission from animal sources; importantly, cross-transmission is known to be highly prevalent among these hosts [89]. Cross-transmission of oxyurid in bats may occur via occasional feeding on rodent and avian species [7, 90].

## 5. Conclusions

In conclusion, the current study contributes to the understanding of GI parasites and their roles in disease according to their feeding habits. The study also suggests that compared to the frugivorous bats, the insectivorous bats have a wide and complex behavioral and ecologic landscape including the selection of insect diets, water bodies, and sharing of an ecosystem with other vertebrates which are critical for transmission of the parasitic species. Based on the wide diversity of parasite richness and parasitic concurrency measured by the prevalence rates, we suggest that GI parasitism might be a threatening factor in the insectivorous bats in the current study area. However, further detailed molecular and epidemiologic studies are essential to identify the species, to assess their pathology, and to analyze their host specificity to clarify their roles in threatening the bats.

## Figures and Tables

**Table 1:** Parasitic species, their concurrency, and prevalence in the frugivorous and insectivorous bats in Southcentral Nepal. Fisher's exact test (two-tailed) was used to calculate the *p* values by comparing the prevalence rates of different parasitic species or groups between the frugivores and insectivores.

Parasitic infections	Frugivores (*N*1 = 30)	Insectivores (*N*2 = 30)	Overall (*N* = 60) prevalence (*n* × 100/*N*)	*p* values
	Prevalence (*n* × 100/*N*1)	Prevalence (*n* × 100/*N*2)		
*Entamoeba* sp.	12 (40%)	9 (30%)	21 (35%)	*p* < 0.0001
*Eimeria* spp.	4 (13.3%)	25 (83.3%)	29 (48.3%)	
*Isospora* sp.	0	5 (16.7%)	5 (8.3%)	
*Cryptosporidium* sp.	0	3 (10%)	3 (5%)	
*Giardia* sp.	0	1 (3.3%)	1 (1.7%)	
**Total Protozoa**	**14 (46.7%)**	**28 (93.3%)**	**42 (70%)**	
Ascarid spp.	0	5 (16.7%)	5 (8.3%)	*p* < 0.0001
*Hymenolepis* spp.	6 (20%)	15 (50%)	21 (35%)	
Strongyle	0	17 (56.7%)	17 (28.3%)	
Oxyurid sp.	0	2 (6.7%)	2 (3.3%)	
*Strongyloides* sp.	0	5 (16.7%)	5 (8.3%	
Capillarid sp.	0	1 (3.3%)	1 (1.7%)	
**Total Helminths**	**6 (20%)**	**24 (80%)**	**30 (50%)**	
Single infection	14 (46.7%)	4 (13.3%)	18 (30%)	*p* < 0.0001
Mixed infection	4 (13.3%)	26 (86.7%)	30 (50%)	
Duplet infection	4 (13.3%)	7 (23.3%)	11 (18.3%)	
Triplet infection	0	8 (26.7%)	8 (13.3%)	
Quadruplet infection	0	9 (30%)	9 (15%)	
Pentuplet infection	0	2 (6.7%)	2 (3.3%)	

## Data Availability

All data generated or analyzed during this study are included within this article.

## Supplementary Materials

Supplementary Materials Supplementary file 1: patterns of parasitic species in frugivorous and insectivorous bats.
