# Supplementary material for: Prevalence of Gastrointestinal Parasites in the Frugivorous and the Insectivorous Bats in Southcentral Nepal
Source: J Parasitol Res. 2020 Dec 12;2020:8880033. doi: 10.1155/2020/8880033 (PMC7752302; doi:10.1155/2020/8880033)
Supplement: Supplementary Materials — Supplementary file 1: patterns of parasitic species in frugivorous and insectivorous bats. [file 8880033.f1.docx]

**Supplementary file 1.** Patterns of parasitic species in frugivorous and insectivorous bats.

| **Pattern of Infection** | **Frugivorous bat (N1=30)** | **Insectivorous bat (N2=30)** |
| --- | --- | --- |
| Single Infection | *Entamoeba*: 8  *Eimeria*: 2  *Hymenolepis*: 4 | *Eimeria*: 3  *Hymenolepis*: 1 |
| Double Infection | *Eimeria* + *Entamoeba*: 2  *Entamoeba* + *Hymenolepis*: 2 | *Eimeria* + *Entamoeba*: 3  *Eimeria +* Strongyle:1  *Eimeria* + *Hymenolepis*: 2  Strongyle + *Hymenolepis*: 1 |
| Triple Infection | **-** | *Eimeria* + *Entamoeba* + Strongyle: 2  *Eimeria* + Strongyle + *Hymenolepis*: 3  *Eimeria* + *Strongyle* + *Strongyloides*: 1  *Eimeria* + *Strongyle* + Ascarid: 1  *Eimeria* + *Hymenolepis* + Ascarid: 1 |
| Quadruplet Infection | **-** | *Entamoeba* + Capillarid + Strongyle + *Strongyloides*:1  *Eimeria* + Strongyle + *Strongyloides* + *Hymenolepis*:1  *Entamoeba* + *Strongyloides* + *Hymenolepis* + Ascarid: 1  *Eimeria* + *Giardia* + *Cryptosporidium* + *Strongyloides*: 1  *Eimeria* + *Isospora* + Strongyle + *Hymenolepis*: 2  *Isospora* + Strongyle + *Hymenolepis* + Oxyruid: 1  *Eimeria* + *Cryptosporidium* + Strongyle + Ascarid:1  *Eimeria* + *Isospora* + *Entamoeba* + *Hymenolepis*:1 |
| Pentuplet Infection | **-** | *Eimeria* + *Isospora* + Oxyruid + Strongyle + *Hymenolepis*: 1  *Eimeria* + *Cryptosporidium* + *Entamoeba* + Strongyle + Ascarid: 1 |
